# Immunological analysis of phase II glioblastoma dendritic cell vaccine (Audencel) trial: immune system characteristics influence outcome and Audencel up-regulates Th1-related immunovariables

**DOI:** 10.1186/s40478-018-0621-2

**Published:** 2018-12-05

**Authors:** Friedrich Erhart, Johanna Buchroithner, René Reitermaier, Katrin Fischhuber, Simone Klingenbrunner, Ido Sloma, Dror Hibsh, Renana Kozol, Sol Efroni, Gerda Ricken, Adelheid Wöhrer, Christine Haberler, Johannes Hainfellner, Günther Krumpl, Thomas Felzmann, Alexander M. Dohnal, Christine Marosi, Carmen Visus

**Affiliations:** 1 Institute of Neurology, Medical University of Vienna, Währinger Gürtel 18-20, 1097 Vienna, Austria; 2 Department of Neurosurgery, Medical University of Vienna, Währinger Gürtel 18-20, 1097 Vienna, Austria; 3 University Clinic for Neurosurgery, Kepler University Hospital, Johannes Kepler University, Wagner-Jauregg-Weg 15, 4020 Linz, Austria; 4 Activartis Biotech GmbH, Wilhelminenstraße 91/IIf, 1160 Vienna, Austria; 5 Systems Bio Medicine Lab, Bar Ilan University, 5290002 Ramat Gan, Israel; 6 Laboratory for Tumor Immunology, CCRI St. Anna Kinderkrebsforschung, Zimmermannplatz 10, 1090 Vienna, Austria; 7 Clinical Division of Medical Oncology, Medical University of Vienna, Währinger Gürtel 18-20, 1097 Vienna, Austria

**Keywords:** Glioblastoma multiforme, Dendritic cell, Cancer immunotherapy, Immunology, ELISPOT

## Abstract

Audencel is a dendritic cell (DC)-based cellular cancer immunotherapy against glioblastoma multiforme (GBM). It is characterized by loading of DCs with autologous whole tumor lysate and in vitro maturation via “danger signals”. The recent phase II “GBM-Vax” trial showed no clinical efficacy for Audencel as assessed with progression-free and overall survival in all patients. Here we present immunological research accompanying the trial with a focus on immune system factors related to outcome and Audencel’s effect on the immune system. Methodologically, peripheral blood samples (from apheresis before Audencel or venipuncture during Audencel) were subjected to functional characterization via enzyme-linked immunospot (ELISPOT) assays connected with cytokine bead assays (CBAs) as well as phenotypical characterization via flow cytometry and mRNA quantification. GBM tissue samples (from surgery) were subjected to T cell receptor sequencing and immunohistochemistry. As results we found: Patients with favorable pre-existing anti-tumor characteristics lived longer under Audencel than Audencel patients without them. Pre-vaccination blood CD8+ T cell count and ELISPOT Granzyme B production capacity in vitro upon tumor antigen exposure were significantly correlated with overall survival. Despite Audencel’s general failure to induce a significant clinical response, it nevertheless seemed to have an effect on the immune system. For instance, Audencel led to a significant up-regulation of the Th1-related immunovariables ELISPOT IFNγ, the transcription factor T-bet in the blood and ELISPOT IL-2 in a dose-dependent manner upon vaccination. Post-vaccination levels of ELISPOT IFNγ and CD8+ cells in the blood were indicative of a significantly better survival. In summary, Audencel failed to reach an improvement of survival in the recent phase II clinical trial. No clinical efficacy was registered. Our concomitant immunological work presented here indicates that outcome under Audencel was influenced by the state of the immune system. On the other hand, Audencel also seemed to have stimulated the immune system. Overall, these immunological considerations suggest that DC immunotherapy against glioblastoma should be studied further – with the goal of translating an apparent immunological response into a clinical response. Future research should concentrate on investigating augmentation of immune reactions through combination therapies or on developing meaningful biomarkers.

**Electronic supplementary material:**

The online version of this article (10.1186/s40478-018-0621-2) contains supplementary material, which is available to authorized users.

## Introduction

Glioblastoma multiforme (GBM) is the most frequent and most aggressive form of brain cancer [30]. Therapeutic options are limited. Currently, the standard first line treatment of GBM is maximum surgical resection followed by chemotherapy (temozolomide) and radiotherapy. Immunotherapy is a novel treatment hope currently under investigation [27]. Immunotherapeutic strategies being tested in clinical trials include checkpoint inhibitors, peptide vaccines and Dendritic Cell (DC)-based vaccines [24]. Given their critical role in guiding anti-cancer immune reactions, deploying DCs against neoplastic cells seems especially plausible [20]. A number of DC-based vaccines are currently undergoing clinical development [18]. Feasibility and safety of DC-based immunotherapeutic approaches have been proven repeatedly [12, 16, 23]. A survival benefit could, however, not yet be established in clinical trials [18] – despite recent reports of encouraging interim results [16].

The Austrian “GBM-Vax” consortium performed a phase II clinical trial with “Audencel”, an autologous DC-based cancer vaccine. Patient DCs were charged with autologous tumor lysis material. DCs were then matured in vitro via immunological “danger signals” (Lipopolysaccharides and Interferon gamma, IFNγ) and injected into inguinal lymph nodes. This maturation step as well as the fact that loading with autologous whole tumor lysate generates a personalized vaccine should in theory mean a technically advanced, promising concept [6, 11]. But the trial based on the Audencel technique failed to show clinical efficacy (see Buchroithner et al. [2]) when assessing progression-free and overall survival in all patients. One potential reason identified by Buchroithner et al. for Audencel’s failure is the temporal proximity to the concomitant chemotherapy weakening the immune system.

**Fig. 1:**
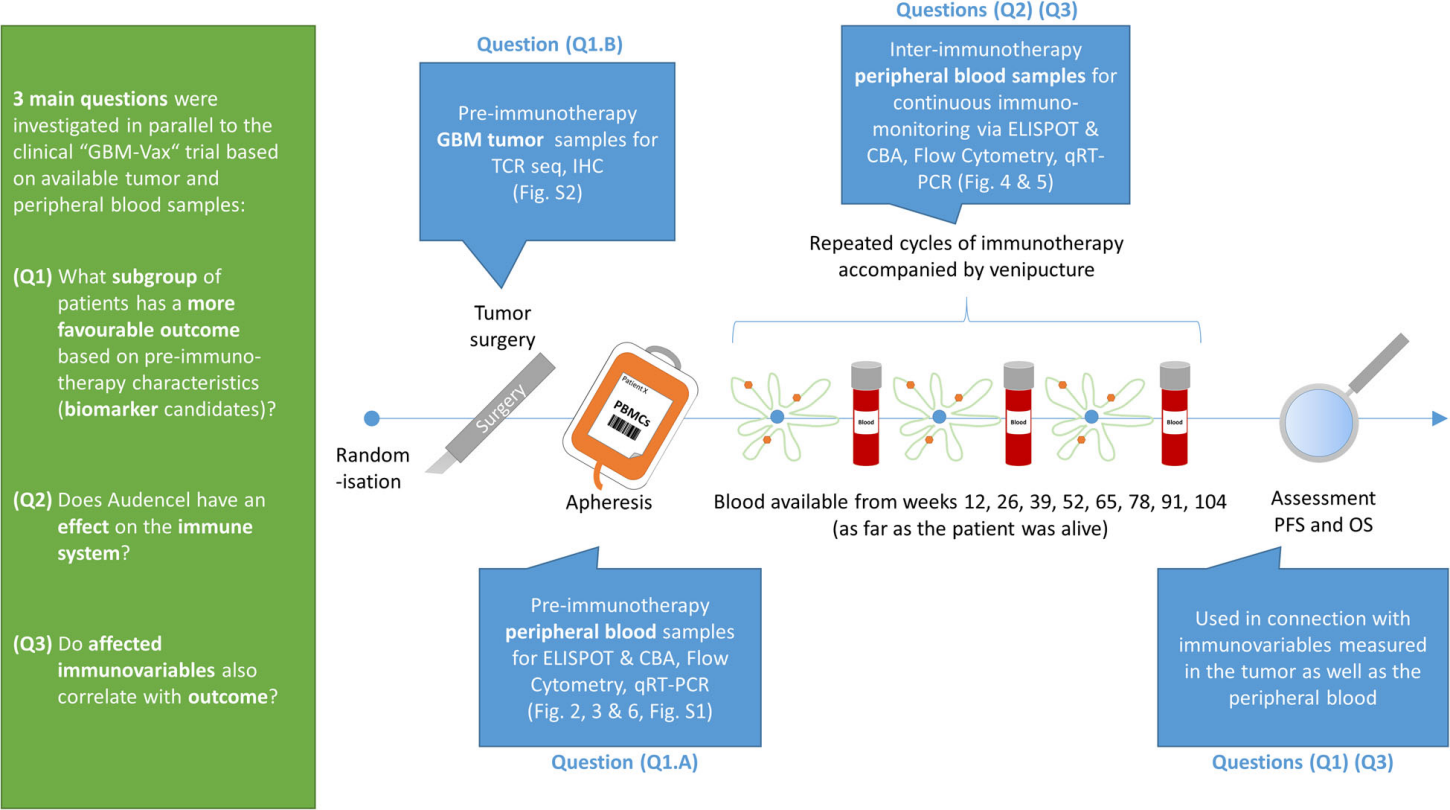
Research questions, samples and techniques

Here, we present the results of immunological research accompanying the trial. We analyzed both, the peripheral blood (from apheresis or venipuncture) as well as the tumor tissue (from surgery) through an array of complementary methods (see Fig. 1a and Materials and Methods) that characterize the immune system via “immunovariables”. The main intention of the here presented investigation was to understand the role of the immune system – measured before, during and after DC vaccination – for DC immunotherapy against glioblastoma.

This meant finding answers to three questions: (Q1) What subgroup of patients based on *pre*-immunotherapy characteristics of the blood and of the tumor might have a more favorable outcome under Audencel and what are hence possible future biomarker candidates? (Q2) Even if there was no clinical response of patients to Audencel, did it have an effect on the immune system? (Q3) Do immune system variable levels *post* Audencel-application also correlate with clinical outcome? To answer question (Q1) we assessed immune system variables in apheresis or tumor samples *before* immunotherapy and correlated them with survival. For question (Q2) we studied immune *dynamics* via measuring blood variables after every cycle of vaccination. Question (Q3) was then approached via combining immunovariable levels post Audencel-treatment with survival measures.

## Materials and methods

### Scientific concept and overview

The present work analyses the clinical trial “GBM-Vax” (NCT01213407/EudraCT2009–015979-27) immunologically. The study was approved by the Upper-Austrian ethical review committee (TRX2/P-II-018). All patients gave written informed consent also to concomitant immunological research.

Various complementary immunological methods were applied to blood and tumor tissue of the participating patients: Functional immune-capabilities were measured in blood samples via an autologous system: the enzyme-linked immunospot (ELISPOT) assay [4, 14]. It quantifies the potency of tumor-charged DCs to stimulate T cells in vitro via registering IFNγ and Granzyme B (GranzB) produced in the co-culture. This replicates the expected functional interplay between DCs and T cells in the patient’s lymph nodes. Further cytokines released in vitro were measured via cytokine bead assays (CBA) of the ELISPOT co-culture supernatant. ELISPOT assays were performed from blood before, during and after Audencel treatment.

In addition, we quantified blood immune cell populations at these time-points via flow cytometry of surface markers. Polarization towards a T helper 1 (Th1), T helper 2 (Th2), T helper 17 (Th17) or regulatory T cell (Treg) phenotype was measured via qRT-PCR of characteristic transcription factors [32, 35, 37, 38].

Tumor tissue was studied via sequencing of tumor-resident T cell receptors (TCR clonotyping) [7] and immunohistochemistry (IHC) of tumor-resident immune cells.

To take technical peculiarities of different methods into account, analysis was done for each method separately. Across all methods, for the stratification of patients into groups with a “high” or “low” immunovariable level, the respective median was taken as the threshold.

Fig. 1 and Additional file 1: Tables S1, S2, S3 and S4 give an overview of all techniques applied. For further details on Methods see Additional file 1: Supplementary methods.

#### Blood: ELISPOT

PBMCs and DCs charged with tumor lysate were co-cultured in MAHAS4510 Millipore 96-well plates (IFNγ or GranzB) and spots, representing single reactive cells, were quantified via the ImmunoSpot® S6 Core ELISPOT-analyzer (C.T.L., Shaker Heights, OH, US). To increase validity, two sources of lysate-loaded DCs were used: the previously frozen Audencel vaccine that had been administered to the patient as well as DCs freshly loaded with lysate. Not all available samples could be successfully used for all ELISPOT experiments due to technical challenges inherent to the ELISPOT technique [3]. In that case, the maximum number of samples with a reliable readout was used for statistics.

#### Blood: CBA

Supernatant from the ELISPOT GranzB plate was collected from the co-culture of PBMCs with or without exposure to either DCs unloaded or loaded with tumor proteins (in triplicates). The supernatant was incubated with CBA Human T helper Th1/Th2/Th17 capture beads (BD Bioscience), washed and subjected to analysis on a BD LSR-II cytometer together with cytokine protein standards.

#### Blood: Cell surface marker analysis

Mononuclear cells (PBMCs) collected at the various time points were measured on a BD LSR-II cytometer (BD Bioscience, Heidelberg, Germany) for the following marker panels: T cells (CD3/8/4/27/28/45RA/CCR7), B cells (ID, 24/19/38/27/20/3), NK/NKT cells (CD94/314/HLADR/16/56/8/3), Tregs/Th17 (161/25/4/45RA/127/8/3), Activated/Modulatory cells (4/38/HLADR/8/3/274) and MDSCs (CD11b/HLADR/33/15/14).

#### Blood: qRT-PCR

IFNγ, TBET, IL4, GATA3, IL10, FOXP3, TGFβ, IL17A, RORγT and the housekeeping gene 18-ribosomal RNA (18rRNA) were measured via the Taqman® 7500 Violet PCR system (Applied Biosystems) in the PBMC population from leukocyte apheresis or venipuncture. Readouts of mRNA PCR were assessed as single markers or as a combination of markers specific for the Th1/Th2/Th17/Treg polarization of the immune system (Th1: Tbet+IFNγ, Th2: IL4 + GATA3, Th17: IL17 + RORγt, Treg: IL10 + FOXp3 + TGFβ) – absolute (sum) as well as relative (polarization-specific factors in relation to all factors measured).

#### Tumor: TCR sequencing

Complementary Determining Regions 3 (CDR3) were amplified by Adaptive Biotechnologies (Seattle, WA) using the immunoSEQ assay. A multiplex PCR system was used to amplify CDR3 sequences from genomic DNA extracted from GBM tumor samples. The ImmunoSEQ approach generated an amplification fragment identifying the VDJ region spanning each unique CDR3. Amplicons were sequenced using the Illumina HiSeq platform. Computational adjustments were used to correct for the primer bias common to multiplex PCR reactions. Raw sequence data were filtered and normalized to the amount of DNA used.

#### Tumor: Immunohistochemistry

Tumors were stained with antibodies against CD45, CD3, CD4, CD8, CD45RO, CD31. Slides were evaluated in central tumor areas, perivascular and at the tumor-infiltrative margin with a Zeiss Axioplan microscope. Staining intensity was evaluated semi-quantitatively by two reviewers as sparse/moderate/dense according to three representative fields of vision.

### Statistics

#### Blood: Statistical analysis of question (Q1.A)

Blood collected before, during (=repeatedly after vaccination) and within days after the last Audencel treatment was subjected to four immunological assays (ELISPOT, CBA, flow cytometry, qRT-PCR). To explore an association with survival the same process was followed for all techniques: The Pearson correlation coefficient was calculated. Then, to examine potential usage as a future biomarker, Kaplan-Meier curves were plotted based on stratifying patients into groups with variable levels above or below the variable median. We considered only those variables as relevant that significantly separated Kaplan-Meier curves or had at least a significant Pearson correlation with survival. No multiple testing corrections were applied due to the exploratory nature of the investigation. To integrate pre-treatment blood markers associated with survival in the single-parameter analysis described above, we combined the 9 most relevant of them into one variable (“high/low” anti-tumor immunity) based on a scoring system. All available blood samples taken before start of immunotherapy were used for that analysis (apheresis + venipuncture from day 1). The 9 variables originated from ELISPOT (IFNγ, GranzB), flow cytometry (CD8+, Tregs, Monos) and qRT-PCR (Th1, Tregs). To control for ELISPOT variability, ELISPOT results from both DC sources (frozen vaccine and freshly loaded, see methods) were part of the score. For every value above the respective median, 1 point was awarded. If not all measurements were available for a patient, the patient was still included in the scoring system – with 0 points for the missing method. A total score of at least 5 points resulted in classifying the patient as having “high” immune-capabilities (Additional file 1: Figure S3).

#### Tumor: Statistical analysis of question (Q1.B)

Entropy (a diversity measure), clonality, Gini index (an unevenness measure), maximum frequency, fraction of productive reads and fraction of unique productive reads were calculated after crossing CDR3 sequences with public TCR-data. Then Kaplan-Meier analyses were performed.

#### Blood: Statistical analysis of questions (Q2 + Q3)

For the assessment of Audencel’s effect on the immune system, the repeated measurement of blood variables during immunotherapy was connected to the number of vaccines applied (Pearson correlation). Post-vaccination levels were defined as the arithmetic mean of all measurements after the first vaccination (to even out time kinetics and differences in sample availability). Relative response strength was calculated as the difference between post- and pre-vaccination levels.

For all statistical tests, *p*-values < 0.05 were considered significant. Software used: SPSS 23 and GraphPad Prism 6.

## Results

### (Q1-Q3) sample availability varied – In the treatment group up to 43 samples could be analyzed

To prepare the intended immunological investigation of the Audencel clinical trial, we started with mapping the availability of patients and samples for research. While the concomitant clinical paper by Buchroithner et al. [2] had to follow stringent regulatory criteria (e.g. age) for formal efficacy assessment and could analyse 34 vaccinated patients, for the experimental immunology research described here, it was possible to analyse 43 patients (with available samples) that were vaccinated in the course of the clinical trial.

An overview of all samples processed successfully is given in Additional file 1: Table S1. For the four intended blood-based research methods and the two intended tumor tissue-based research methods, sample availability varied considerably. The highest number of blood-based samples (43 prior to Audencel treatment, 34 during Audencel treatment cycles, 7 prior to control treatment) was reached for flow cytometry and qRT-PCR of immune cell markers. A lower number of blood-based samples was reached for ELISPOT (32 prior to Audencel, 22 during Audencel, 4 prior to control) and CBA (36 prior to Audencel, 26 during Audencel, 4 prior to control).

For tumor-based methods, more control samples were available but at the same time fewer treatment samples: For TCR sequencing we arrived at 23 samples prior to Audencel and 15 prior to control. For IHC it was 11 prior to Audencel and 14 prior to control. Tumor specimens were only seized prior to the respective treatment but not at later time points.

Different sample sources were a cause for the variability of samples measured across immunological methods. Additionally, technical limitations (e.g. amount of material needed for a test) restricted full usage of available samples.

### (Q1.A) Pre-vaccination blood: CD8+ cells and ELISPOT response correlated with OS under Audencel

As the first actual research step, we wanted to elucidate a possible impact of *pre-existing* immune system differences across patients on clinical outcome. Thus, we studied immunovariables *before* DC immunotherapy (Additional file 1: Table S2) and related them with outcome parameters. In that investigation, we began with measuring blood-based variables that characterize the state of the immune system phenotypically and functionally. ELISPOT and CBA assessed the potency of anti-tumor immune reactions while flow cytometry and qRT-PCR registered populations and polarizations of immune cells in the blood (details see Methods). With the help of these techniques we found several associations: pre-vaccination levels of peripheral blood CD8+ T cells, ELISPOT GranzB production, ELISPOT IFNγ production, blood monocytes, and Th1-related blood transcription factors were associated positively with OS. Pre-vaccination Treg levels in the blood were associated negatively with OS. 

**Fig. 2:**
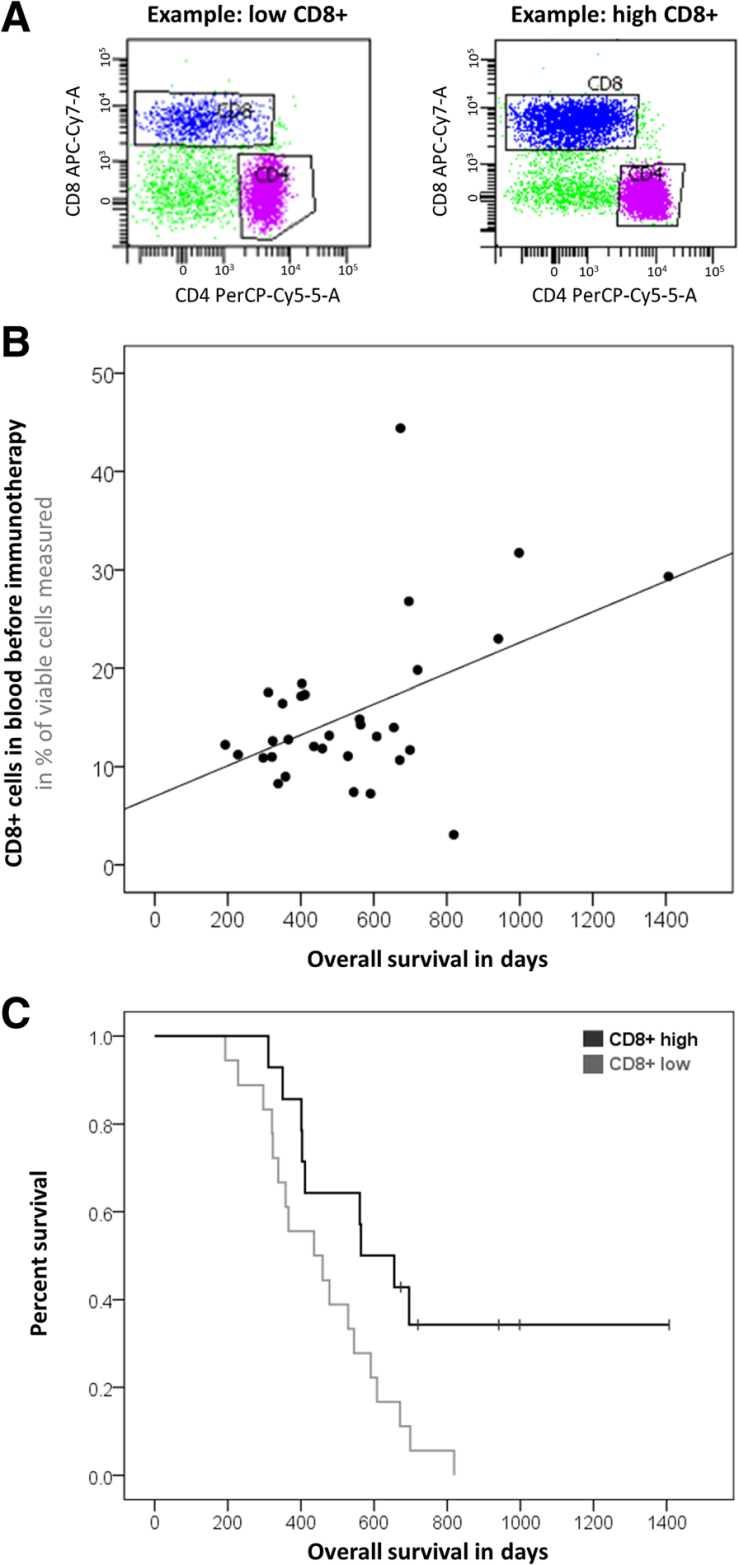
Pre-Audencel blood CD8+ count. **a** Example of “low” and “high” CD8+ count. **b** CD8+ count correlated with survival (*p* = 0.005, *n* = 32). **c** Significant survival curve separation (*p* = 0.018, n = 32)

These findings are based on the following evidence: The percentage of CD8+ T cells in the blood of Audencel patients significantly correlated with OS (Pearson correlation, *p* = 0.005, Fig. 2b). Patients with “high” levels of CD8+ cells (above the median) already before immunotherapy lived significantly longer under Audencel than patients with pre-therapy CD8+ levels below the median (Kaplan-Meier analysis, *p* = 0.018, Fig. 2c).

**Fig. 3:**
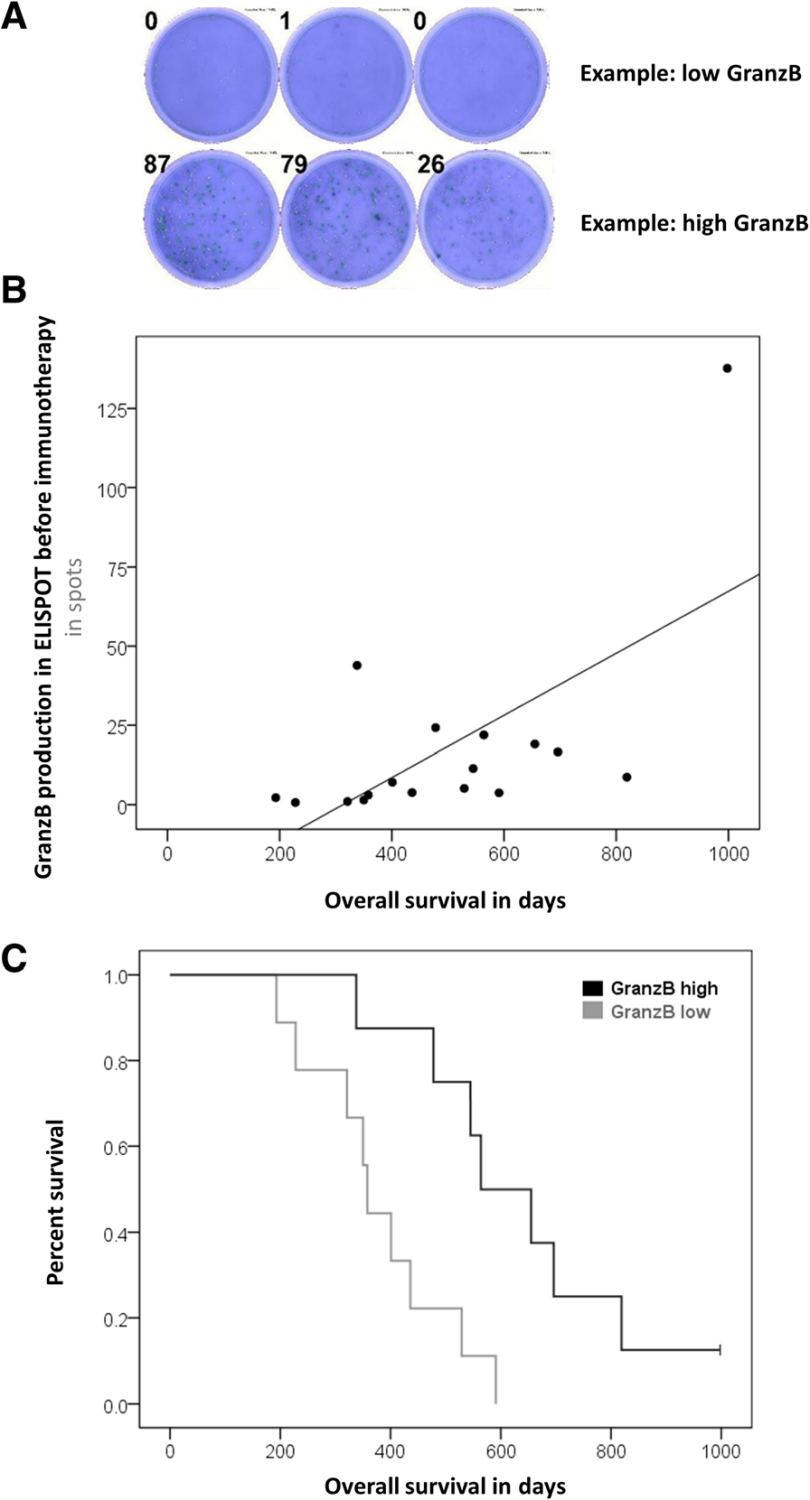
Pre-Audencel ELISPOT Granzyme B. **a** Example readout. **b** Granzyme B correlated with survival (*p* = 0.007, *n* = 17). **c** Significant survival curve separation (*p* = 0.006, n = 17)

Similarly, patients with pre-existing immunity to autologous tumor antigens lived longer under Audencel (Fig. 3): GranzB production in tumor antigen-specific ELISPOT assays correlated significantly with OS (Pearson correlation, *p* = 0.007, Fig. 3b). The patient group with GranzB production above the median also lived significantly longer (Kaplan-Meier analysis, *p* = 0.006, Fig. 3c). For ELISPOT IFNγ, analogous observations were made for progression-free survival (PFS, Pearson correlation: *p* = 0.040, Kaplan-Meier analysis: *p* = 0.003). In terms of OS, for ELISPOT IFNγ a significant correlation was registered (Pearson correlation, *p* = 0.037) but for the separation of survival curves only a trend without reaching significance could be seen (Kaplan-Meier analysis, *p* = 0.615). Further, the higher the pre-existing blood monocyte count, the longer was OS under Audencel (Pearson correlation, *p* = 0.005, Additional file 1: Figure S1A). Again, also survival curves were separated significantly (Kaplan-Meier analysis, *p* = 0.028, Additional file 1: Figure S1B). Regulatory T cells (Tregs), on the other hand, were inversely correlated with OS: the lower the pre-vaccination levels of Tregs, the longer the survival (Pearson correlation, *p* = 0.0001). Treg-separated Kaplan-Meier curves showed a trend but no significance (*p* = 0.528). The relative fraction of Th1-related transcription factors (Tbet+IFNγ) correlated positively with OS (Pearson correlation, *p* = 0.020), but in Kaplan-Meier analysis only a trend was seen (*p* = 0.241).

Summing up, IFNγ, Th1-factors and Tregs were associated with OS in Pearson correlation testing – GranzB, monocytes and CD8+ cells also reached significance when used for Kaplan-Meier curves. For further pre-vaccination blood results see Additional file 1: Table S2: E.g. T cells, B cells, NK cells, granulocytes and sub-populations of them were not associated with survival when measured pre-vaccination. For control patients, none of the variables showed a significant association with survival in Kaplan-Meier analyses (data not shown) – the sample size for control patients was, however, considerably low (see above).

### (Q1.B) Pre-vaccination tumor: T cells were associated with a non-significant trend towards longer OS under Audencel

As the next step, we extended our investigation of pre-vaccination parameters from the blood to the tumor. Hence, while the previous analyses focused on peripheral blood immune cells, we here studied tumor-resident immune cells. This time, sufficient material was available for both, the treatment (TCR: *n* = 23; IHC: *n* = 11) and the control group (TCR: *n* = 15; IHC: *n* = 14).

First, we assessed the repertoire of T cell receptors in GBM tissue via TCR sequencing. We observed that GBM tissue showed a more heterogeneous but also narrower TCR repertoire than blood samples (data not shown). Variables measuring TCR diversity in the tumor (Gini index, clonality, clonal evenness, entropy) were not associated with clinical outcome.

Another TCR sequencing-based analysis looked at the impact of general T cell abundance in the tumor. Given that blood data (Fig. 2) had indicated T cell levels might affect survival, we assessed whether this was also reflected in the sequencing data.

Therefore, we used the number of productive TCR reads as a proxy for T cell abundance. When selecting patients with productive reads (normalized to total reads) above the median, Audencel-treated patients showed a trend towards longer OS than control patients with the same feature but without reaching significance (*p* = 0.061, Additional file 1: Figure S2A). The abundance of (CD8+) T cells in the blood did not correlate significantly with T cell abundance in the tumor (*p* = 0.898, Additional file 1: Figure S2B).

Furthermore, we used IHC to explore immunological markers in the tumor. We observed that the overall amount of CD8+ cytotoxic T cells in the tumor correlated positively with PFS (Pearson correlation, *p* < 0.001, not shown). Similarly, the overall level of CD45RO+ memory T cells was associated with PFS (Pearson correlation, *p* = 0.017, not shown). Also, the relative amount of microvasculature at the tumor margin (as measured via CD31+ endothelial cells) was related to survival (PFS, Pearson correlation, *p* = 0.046, not shown). However, none of these markers led to a significant separation of survival curves in the Kaplan-Meier analysis (Additional file 1: Table S2). And none of the ICH markers showed an association with OS of Audencel-treated patients. In the control group, neither PFS nor OS were influenced by IHC markers.

### (Q2) Vaccination effects on blood: Audencel stimulated Th1-related functional immunovariables in a dose-dependent manner

**Fig. 4:**
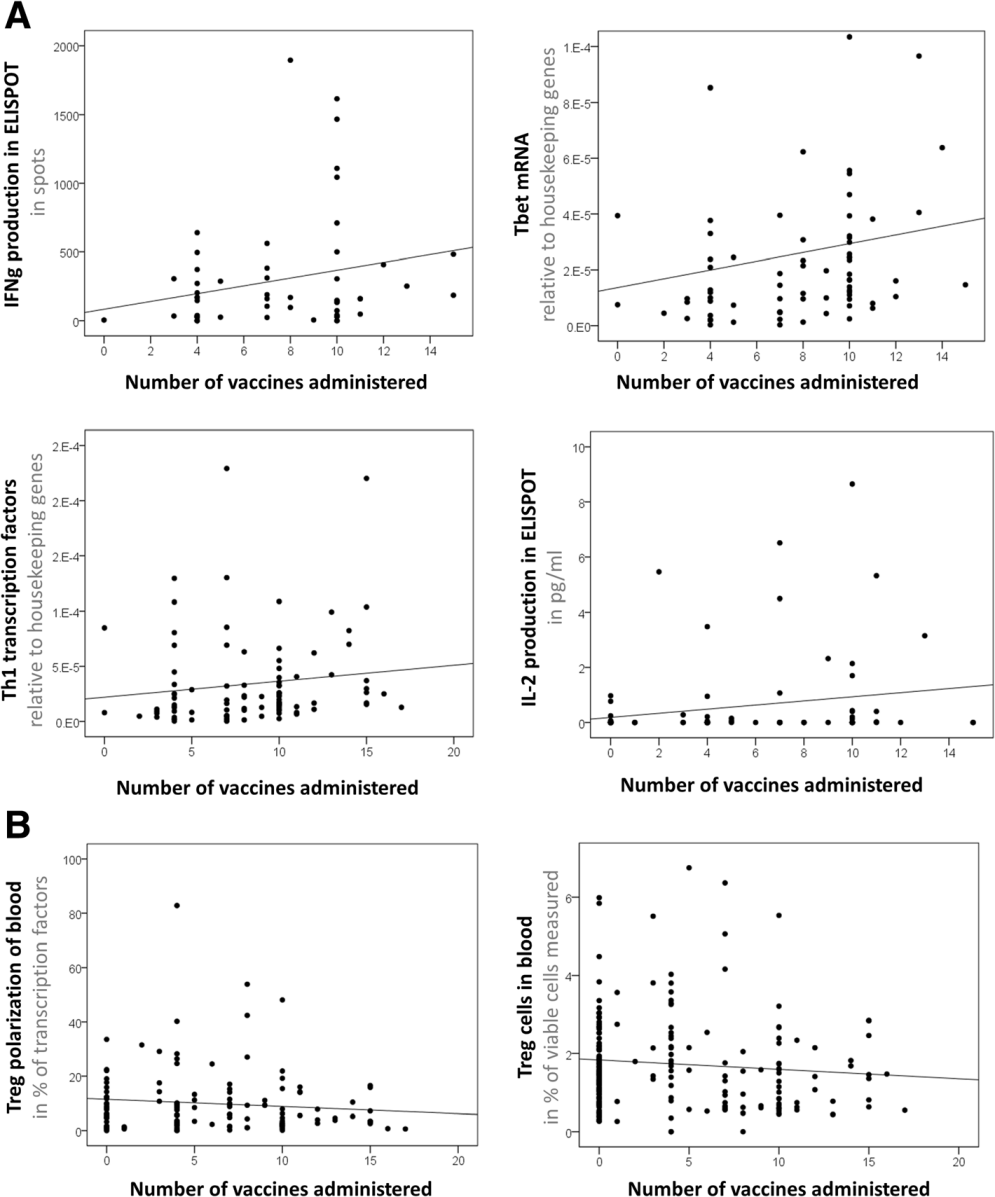
Audencel’s effect on the immune system. **a** Significantly positive correlation between vaccines administered and ELISPOT IFNγ (*p* = 0.038, *n* = 22), Tbet mRNA (*p* = 0.006, *n* = 34), Th1 transcription factors (combined qRT-PCR measurement of Tbet and IFNγ mRNA, *p* = 0.006, n = 34) and ELISPOT IL-2 (*p* = 0.001, *n* = 26). **b** Significantly negative correlation for blood Treg polarization (*p* = 0.036, n = 34) and Treg cells (*p* = 0.034, *n* = 34). The plots depicted in a and b show all available time points from all available patients (*n* = patients)

**Fig. 5:**
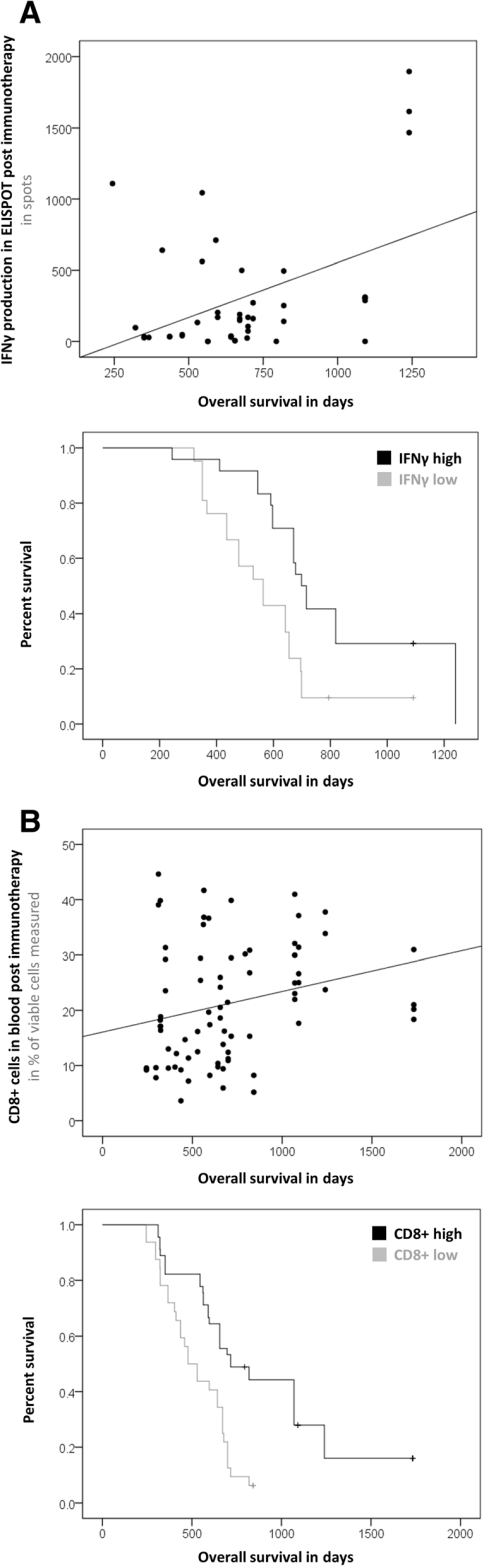
Relevant post-Audencel immunovariables and outcome. **a** ELISPOT IFNγ significantly correlated with OS (*p* = 0.022, n = 22). Patients with “high” ELISPOT IFNγ (above the median) also showed a significantly better OS (p = 0.003, n = 22). **b** CD8+ correlated with OS (*p* = 0.026, n = 34) and separated survival curves significantly (*p* < 0.001, n = 34)

Subsequently, we aimed at studying if Audencel might have effects on the immune system. For that, we registered blood variable levels after each round of DC vaccination and plotted their respective dynamics. We found that IFNγ in ELISPOT assays correlated significantly with the number of vaccines given (Pearson correlation, *p* = 0.038, Fig. 4a). The same holds true for Tbet mRNA levels (Pearson correlation, *p* = 0.006, Fig. 4a) in blood cells (PBMCs). Also, a combined measure of cytotoxic immune responses (mRNA of Th1 transcription factors Tbet and IFNγ) significantly increased upon Audencel administration in a dose-dependent manner (Pearson correlation, p = 0.006, Fig. 4a) – while blood IFNγ mRNA levels alone declined (Pearson correlation, *p* = 0.003, not shown). Moreover, the ELISPOT production capacity of Interleukin-2 (IL-2, Pearson correlation, *p* = 0.001, Fig. 5a) was enhanced with every vaccination and equally the Interleukin-17 (IL-17) production capacity (Pearson correlation, *p* = 0.002, not shown).

When looking at immunovariables with a decrease upon vaccination, we found that the blood Treg polarization declined with every cycle of Audencel treatment (Pearson correlation, *p* = 0.036, Fig. 4b). Also, Treg cells in the blood declined with every vaccination (Pearson correlation, *p* = 0.034, Fig. 4b). Similarly, overall CD4+ cells as well as subsets of them decreased (Additional file 1: Table S3).

Taken together, Audencel treatment seems to functionally skew the immune system towards Th1 reactions in a dose-dependent manner, resulting in an additional stimulation with every additional vaccination.

Immunovariables such as ELISPOT GranzB, CD3+ cells, CD8+ cells, B cells, NK cells, monocytes or granulocytes did not react to Audencel vaccination (Additional file 1: Table S3).

### (Q3) Post-vaccination blood: ELISPOT IFNγ and CD8+ cells were associated with clinical outcome

Given that Audencel apparently altered the immune system, we next wanted to study if *changes* upon Audencel application were directly associated with survival. However, for none of the variables identified in (Q2), the relative strength of the variable response upon vaccination correlated with PFS or OS (not shown).

Subsequently, we looked at the *absolute* immunovariable levels post vaccination because we assumed that the overall effect of vaccination might have an influence on outcome – independent of the relative change. To take the different number of vaccinations given across patients and potential time kinetics into account, we used all available post-vaccination time points (from all available patients; n=patients).

As a result, we noticed that several variables measured after vaccination were indeed connected with clinical outcome: Post-vaccination ELISPOT IFNγ production significantly correlated with OS (Pearson correlation, *p* = 0.022, Fig. 5a) and could significantly separate survival curves (Kaplan-Meier analysis, *p* = 0.003, Fig. 5a). Similarly, post-vaccination CD8+ cell abundance in the blood correlated with OS (Pearson correlation, *p* = 0.026, Fig. 5b) and separated survival curves (Kaplan-Meier analysis, *p* < 0.001, Fig. 5b). Monocyte levels post vaccination showed the same association with OS (Pearson correlation: *p* < 0.001, Kaplan-Meier analysis: *p* = 0.008, not shown). Interestingly, this was also true for activated NK cells (Pearson correlation: *p* = 0.042, Kaplan-Meier analysis: *p* = 0.024, not shown). In an additional analysis that looked at the data from yet another angle (assuming the post-vaccination average of all time points as the relevant overall post-vaccination level), we registered that CD8+B7H1+ cells and CD4+B7H1+ cells correlated significantly with OS (Pearson correlation, *p*<0.001 for both) but could not separate survival curves (Kaplan-Meier analysis, CD8+B7H1+ *p*=0.219, CD4+B7H1+ *p*=0.085). All further post-vaccination results see Additional file 1: Table S4.

### (Q1 + Q2 + Q3) Integration: Patients with “high” immune-capabilities showed better outcome under Audencel

Finally, we studied whether patients with generally “high” anti-tumor immune-capabilities were more likely to benefit from DC vaccination. Our assumption was that the single variables we had found previously could be condensed to one overall illustrative measure. Thus, we integrated prior insights into one parameter via a scoring system. To allow potential future usage as a clinical biomarker, we exclusively used pre-vaccination variables from the blood for that score. Up to 9 points were awarded for individual immunovariables and added up: “high” anti-tumor immune-capabilities were arbitrarily defined as 5–9 points, “low” immune-capabilities arbitrarily as 1–4 points. 1 point each was awarded for high levels (above the median) of Th1 indicators, IFNγ, GranzB, CD8+ cells and monocytes; 0 points were given for high levels of Tregs or low levels of all the other variables mentioned – again relative to the median (Additional file 1: Figure S3). Consequently, 12 of the treatment patients were classified as having “high” and 31 as having “low” capabilities.

**Fig. 6:**
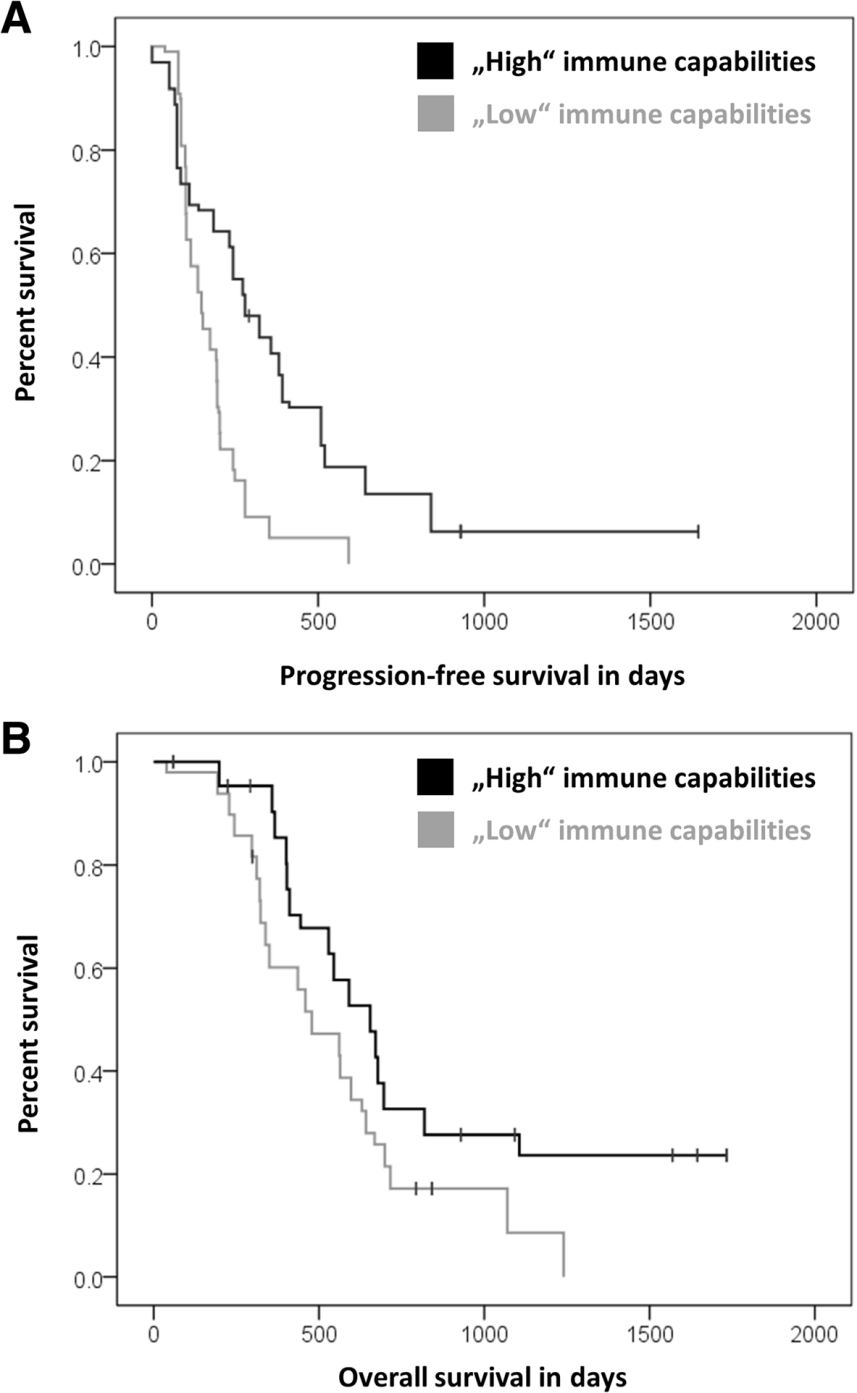
Integration of “high” or “low” blood-based pre-vaccination immune-capabilities with a scoring system. **a** Audencel patients with “high” immune-capabilities have a significantly longer PFS (*p* < 0.001, *n* = 43) and (**b**) OS (*p* = 0.014, *n* = 43)

As a result, we could observe that patients with “high” anti-tumor immune-capabilities had a significantly better outcome in terms of PFS (Kaplan-Meier analysis, *p* < 0.001, Fig. 6a) as well as OS (Kaplan-Meier analysis, *p* = 0.014, Fig. 6b). In the control group, only data from 7 patients were available for this analysis: no association with survival was registered for “high” immune-capabilities (*p* = 0.695, Additional file 1: Figure S4).

Also, we checked whether patients with “high” immune-capabilities before vaccination were the ones that showed altered levels of immunovariables after vaccination. This was not the case. We found no significant difference in all relevant variables after vaccination between patients with “high” or “low” immune system-capabilities before vaccination (Additional file 1: Figure S5). Patients with “high” variable levels after vaccination were not necessarily the same patients that had “high” immune system-capabilities before vaccination.

## Discussion

As essential findings of this immunological analysis of a phase II DC immunotherapy trial (GBM-Vax), we show that Audencel-treated patients with specific immune system characteristics (immunovariables) lived significantly longer than other Audencel-treated patients. We also demonstrate that DC immunotherapy had effects on the immune system. The patient’s immune system and Audencel appear to mutually influence each other.

In response to the research questions initially asked, the following answers can now be given: For question (Q1) – that asked for possible relations of pre-vaccination immune system parameters with outcome – we found that blood CD8+ T cell count above the median was indicative of longer OS. The same holds true for GranzB production in ELISPOT assays as well as the blood monocyte count. Major pre-vaccination biomarker candidates for future studies are thus blood CD8+ cells, blood monocytes and ELISPOT GranzB from blood.

Regarding question (Q2), which aimed at investigating immunological changes upon vaccination, we observed that Audencel DC immunotherapy led to a significant up-regulation of functional Th1-related immunovariables in a dose-dependent manner: IFNγ and IL-2 production in ELISPOT assays and the transcription factor Tbet were enhanced with every vaccination. Conversely, peripheral blood Treg-polarization and the blood Treg population were reduced with every vaccination. Hence, even if no clinical response in terms of survival could be established in the GBM-Vax trial, there is still first indicative evidence of a potential biological effect Audencel exerts on the immune system.

Regarding question (Q3), that deals with the association of post-vaccination variable levels and outcome parameters, we could show that immunovariables measured post vaccination were indeed connected to outcome. Post-vaccination ELISPOT IFNγ, blood CD8+ T cells and monocytes above the median were significantly indicative of longer survival. The relative strength of variable level change (with respect to the pre-vaccination level) was not related to outcome in our studies, however.

When integrating the immunological findings from all the questions asked, the immune parameters ELISPOT IFNγ and blood CD8+ cells stick out and are of special interest: Pre-vaccination ELISPOT IFNγ was associated with OS in the Pearson correlation but not in the Kaplan-Meier analysis. Upon vaccination, ELISPOT IFNγ levels increased significantly. Post-vaccination ELISPOT IFNγ was then also associated with OS in the Kaplan-Meier analysis.

Blood CD8+ T cells above the median were already indicative of longer OS in the pre-vaccination Kaplan-Meier examination. Post-vaccination blood CD8+ cells showed then the same association. Vaccination, however, induced no significant increase of CD8+ levels.

Summing up, if patients had an immune system with “high”, Th1-related anti-tumor capabilities, they had a tendency towards better outcome – independent of whether these capabilities were pre-existing or vaccination-induced.

### Analysis of blood-based variables

For blood-based measurements, sufficient material was available from treatment patients but only a limited amount from control patients. The blood-based analyses therefore focus on the treatment group and show differences *within* that group. Any blood-based biomarker candidate is thus valid for the interpretation of the relative efficacy between different groups of Audencel-treated patients. Deductions regarding the overall efficacy of Audencel relative to the control group cannot be made. As we could therefore not sufficiently study if patients under standard therapy (control patients) with “high” pre-existing blood-based immune-capabilities fare better, literature data is consulted here: While our results revealed blood T cells as relevant, in the literature, blood NK cells are associated with a favourable outcome [21]. Blood levels of IL-10 and CD39 seem to be associated with a dismal prognosis [21]. We found no standard treatment study in the literature that explored blood biomarkers based on the immune response to tumor material in functional ELISPOT assays – hence *tumor antigen-specific functional* testing as we did. Rather, blood-based biomarker research in glioblastoma is mostly focusing on proteins [22]. For instance, Perez-Larraya et al. showed that preoperative IGFBP-2, GFAP, and YKL-40 plasma levels might be indicative of outcome [9]. Future research in bigger standard-treatment patient cohorts as well as immunotherapy cohorts will have to elucidate if e.g. ELISPOT testing can also predict OS in standard-treatment patients or whether the observations are specific for DC immunotherapy.

The blood-based analysis of marker dynamics in response to DC vaccination showed a general up-regulation of immunostimulatory markers (ELISPOT IFNγ, Tbet mRNA, ELISPOT IL-2) in combination with a down-regulation of immunosuppressive markers (blood Treg count and blood Treg polarization). The only counterintuitive observation is that blood IFNγ mRNA also showed a reduction. Further studies in larger samples will have to investigate why. But even when considering the IFNγ mRNA data, altogether, we see a definite skewing of the immune system towards immunostimulation: 1) Tbet mRNA is going up so intensively that Tbet mRNA plus IFNγ mRNA together – as the sum of all Th1-driving transcription factors – still go up significantly. And Tbet is more prototypic for Th1 polarization than IFNγ [15, 26, 28]. 2) Tumor antigen-respondent IFNγ protein production as measured in the ELISPOT assay increases significantly, which is arguably much more specific and functionally relevant than merely blood mRNA levels. 3) The overall pattern of rising immunostimulatory markers and declining immunosuppressive markers in sum speaks for an immunostimulatory effect.

A topic to consider here is the potential impact of Temozolomide chemotherapy. Buchroithner et al. [2] speculate that it could have an influence on outcome. That theory is in line with our observations: indeed, there seems to be an association of anti-tumor immune capabilities and survival. Importantly, the described up-regulation of immunostimulatory markers we observed in response to vaccination is independent of the recovery of the immune system after chemotherapy: Temozolomide administration was continued in parallel to DC vaccination throughout the whole treatment cycle. While under a general recovery of the immune system all cell populations would gain over time, we saw a specific up-regulation of functional response markers (ELISPOT IFNγ & IL-2) and a down-regulation of the immunosuppressive Treg population.

Taken together, the rise in immunostimulatory markers that we see is thus not a phenomenon of chemotherapy-recovery but evidently vaccination-associated. Future longitudinal studies in larger patient cohorts will have to verify the association we registered.

In the specific field of glioblastoma DC immunotherapy, our blood-based immunovariable observations represent an additional piece of evidence when considering prior publications: Yu et al. registered an expansion of antigen-specific CD8+ T cells after DC vaccination when studying 9 patients via HLA-restricted tetramer staining [36]. Yamanaka et al. used ELISPOT testing on 16 vaccinated patients and detected an increase in tumor lysate-reactive CD8+ T cells, which was related to outcome [33]. Liau et al. applied the Alamar blue cytotoxicity assay to 12 vaccinated patients and also observed tumor-specific cytotoxicity [17]. Wheeler et al. performed qPCR of IFNγ mRNA to detect antigen-directed IFNγ production after vaccination of 32 patients and saw a relation of immunoresponse and outcome [31]. Vik-Mo et al. vaccinated 7 patients with a DC immunotherapy (targeting glioblastoma stem-like cells) and registered post-vaccination lymphocyte proliferation in co-cultures with PBMCs loaded with tumorsphere lysate [29]. Everson et al. monitored pSTAT signaling changes in peripheral blood lymphocytes of 28 vaccinated patients and detected an association with outcome [5]. Finally, Jan et al. evaluated PBMCs of 27 vaccinated patients via immunohistochemistry and saw a connection of a low PD-1+/CD8+ ratio and outcome [13].

In comparison to these prior studies, our results are adding novel experimental evidence in the following ways: First, with up to 43 measured vaccinated patients and an integration of phenotypical and functional measures, this study is one of the largest and most comprehensive so far. It confirms, complements and extends prior work. Second, we also investigated pre-vaccination factors while most other groups focused only on vaccination-induced changes. Pre-vaccination factors have the advantage that they might serve as future clinical decision support for selecting those patients most likely to benefit from immunotherapy.

### Analysis of tumor-based variables

For tumor-based research, sample availability was better: we had an adequate amount of material from the control group at our disposal. Consequently, we could include control group data into our investigation. We saw that Audencel-treated patients with a “high” productive ratio (as a proxy for T cell abundance in the tumor) have a non-significant tendency towards better OS than control patients with a “high” productive ratio. IHC-based investigations could only confirm a role of tumor-resident T cells for PFS within the Audencel patient group but not in comparison to the control group. Therefore, we interpret these findings as an indication of a potential general T cell relevance but not as signs for Audencel’s efficacy in a subgroup. Any conclusions regarding the efficacy of Audencel vis-à-vis control patients cannot be made based on this data.

Prior research on the impact of tumor-resident T cells produced conflicting evidence [1, 19, 34]. In the specific context of DC-based immunotherapy, one TCR sequencing-based study found an association between T cells and PFS/OS [10]. From a biological point of view the immune-context of malignancies is known to have an influence on outcome [8].

The observation that blood T cell abundance and tumor T cell abundance did not correlate in our patients might indicate that the overall size of the T cell “pool” is relevant rather than the pre-vaccination location. Further studies are necessary to validate this speculation.

### Evaluation of limitations and strenghts

One important caveat for all results presented here is the multiple testing fallacy, especially when considering the low number of available samples and the wide array of methods applied. This challenge was also faced by all mentioned prior publications that assessed early-stage DC vaccination trials immunologically. In line with them [5, 13, 17, 29, 31, 33, 36] we did not apply further multiple testing corrections, which is why our observations should be seen as primarily exploratory.

Also, sample availability was different depending on the respective method leading to a different set of patients for every method. That is why we focused on method-specific analyses. In the exploratory context of our efforts, we see this as a valid approach.

Overall, it will be necessary to validate the observations we made here in larger patient cohorts. Until then, caution should be exercised when interpreting the presented findings.

When it comes to strengths of the present immunological work, the finding that patients with favorable immune system characteristics might be more prone to beneficial effects from immunotherapy is especially noteworthy. For the specific Audencel technology the research presented here is the first such evidence. In relation to other, prior DC vaccination immunology studies we add further (confirmatory) experimental data from one of the largest patient groups so far. But in any case, the findings presented here do not justify the clinical usage of Audencel yet – not even for patients with favorable immune-capabilities. Instead, we argue for further studies to elucidate how the immunological effects of Audencel might be translated to a measurable clinical effect. One strategy could be the augmentation of DC-based immunotherapies through the combination with immunostimulatory approaches [25] or dose escalation.

A further strength of our study is the identification of easy-to-use pre-vaccination blood parameters that could aid in selecting patients eligible for DC vaccination. For instance, the relation of peripheral blood CD8+ count and survival under Audencel could be a biomarker candidate worthwhile studying further. It can be measured conveniently and might spare patients from undergoing DC vaccination in vain – potentially saving costs and unnecessary efforts. To the best of our knowledge, the data presented here is the first indication for pre-vaccination blood CD8+ count as a biomarker candidate for DC immunotherapy [5, 13, 17, 29, 31, 33, 36].

## Conclusion

In a recent clinical trial, DC immunotherapy with Audencel failed to improve survival. In the concomitant immunological research presented here, we demonstrate that patients with an immune system equipped with favorable pre-existing or post-vaccination anti-tumor capabilities are more likely to live longer under Audencel. Furthermore, Audencel has effects on the immune system despite failure to show clinical efficacy. This indicates that DC immunotherapy against glioblastoma should be studied further – e.g. via investigating combination therapies or via developing meaningful biomarkers.

## Additional file

Additional file 1: Additional supplementary information (Figures, Tables and Materials and Methods). (DOCX 2090 kb)
