# Supplementary material for: Immunological analysis of phase II glioblastoma dendritic cell vaccine (Audencel) trial: immune system characteristics influence outcome and Audencel up-regulates Th1-related immunovariables
Source: Acta Neuropathol Commun. 2018 Dec 5;6:135. doi: 10.1186/s40478-018-0621-2 (PMC6280511; doi:10.1186/s40478-018-0621-2)
Supplement: Supplementary file 1 — Additional supplementary information (Figures, Tables and Materials and Methods). (DOCX 2090 kb) [file 40478_2018_621_MOESM1_ESM.docx]

**Figure S1.** Of the pre-vaccination parameters measured in the peripheral blood, monocyte count also showed a consistent association with survival. (A) The abundance of monocytes (in % of PBMCs) is significantly correlated with OS (p=0.005). (B) Likewise, if the number of monocytes is used to define groups (with the median as a cut-off), then patients with a “high” level of monocytes live significantly longer (p=0.028).

**A**

**
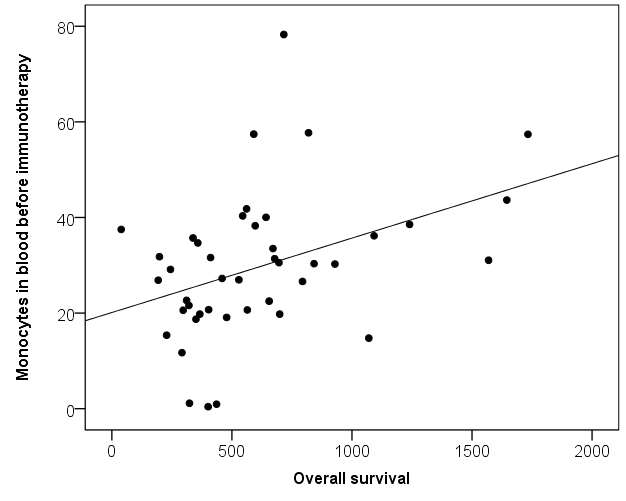
**

**B**

**
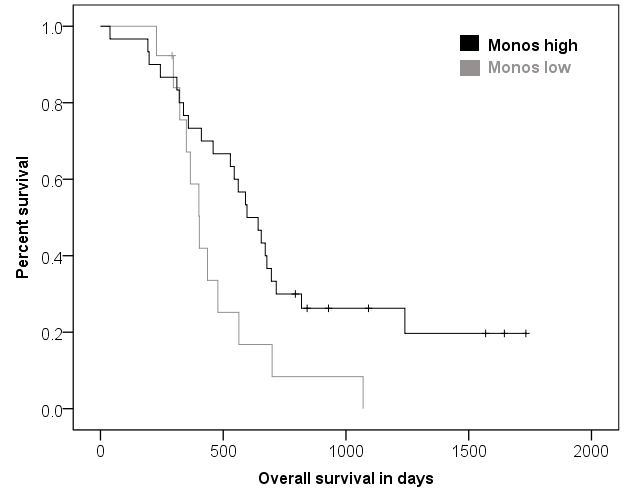
**

**Figure S2.** Results of tumor tissue analysis in search for potential biomarker candidates. Results from the TCR sequencing analysis. (A) Of all markers measured with TCR sequencing, we identified the number of productive reads – a proxy for T cell abundance – as potentially relevant: if only patients with "high" productive reads are selected (treatment: n=15, control: n=10), there is a trend towards longer survival in Audencel-treated patients as opposed to control patients (p=0.06). (B) There is no significant correlation between the abundance of CD8+ cells in the peripheral blood and the abundance of T cells in the tumor (as measured via TCR productive reads, n=23, p=0.898).

**A**

**B**

**Figure S3.** (A) Definition of “high” and “low” immune system capabilities at baseline (before immunotherapy). Pre-vaccination blood variables that showed impact on OS or PFS in single variable analyses were combined. “High” immune system capabilities were defined by the combination of levels above the respective median of the following variables: IFNγ (ELISPOT), GranzB (ELISPOT), CD8 (PBMC flow cytometry), Th1 (qRT-PCR proportion of Tbet and IFNγ mRNA), monocytes (PBMC flow cytometry). Given ELISPOT variability, for the ELISPOT variables both measurement methods (with frozen Audencel vaccine or with freshly-loaded DCs) were part of the scoring system. “Low” immune system capabilities were defined by a level of Tregs above the median or levels of all the other variables mentioned below the median. 58.3% of treatment patients had “low” immune system capabilities before immunotherapy and 41.7% “high” immune system capabilities according to our definition above. For the control group (where only 7 patients could be analyzed) the respective numbers were 42.9% and 57.1%. (B) Depiction of the single markers measured and how the scores for the patients were calculated.

**A**


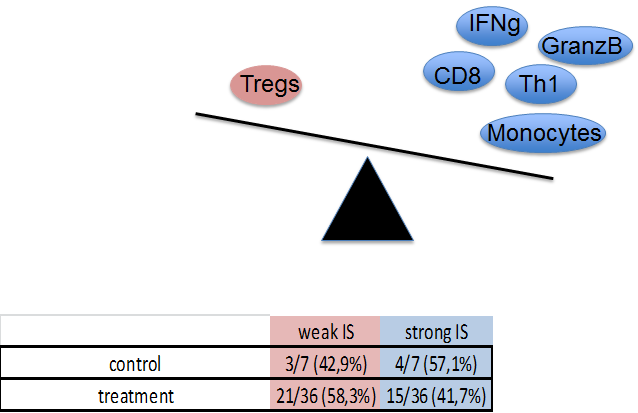


**B**

**Figure S4**. Analysis of patients with “high” or “low” immune capabilities in the control group. As for control patients no immunomonitoring was initially intended in the clinical trial protocol, this analysis could only be performed for 7 control patients. No significant difference in overall survival could be observed (p=0.695, n=7).
Green = “high” immune capabilities, blue = “low” immune capabilities.

**
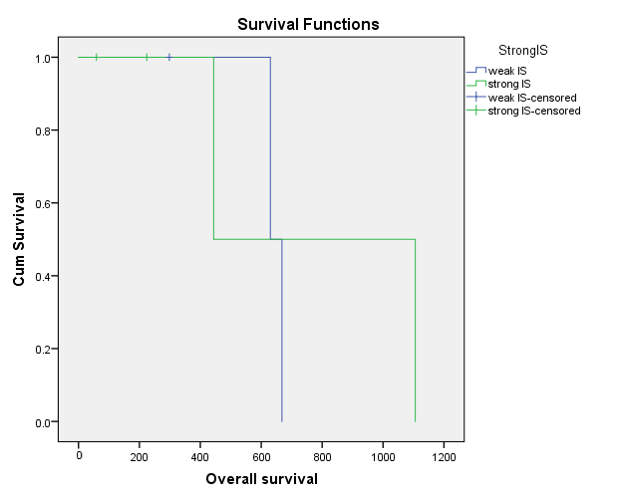
**

**Figure S5.** Analysis of the strength of the immune system prior to vaccination and the possible influences on immunovariable levels after vaccination. (A) Depiction of major variables with a dose-dependent increase upon vaccination. (B) Depiction of major variables where the post-vaccination level is associated with survival. There is no difference in the post-vaccination level of all variables in (A) and (B) based on whether immune system capabilities were “high” or “low” before the vaccination. The pre-vaccination strength of the immune system has apparently no influence. Note that in (B) cell measurement was done in different flow cytometry setups which is why cell numbers do not add up.

**A**

**B**

**Table S1.** Overview of all patient material analyzed from the treatment as well as the control group. Tumor material was only available before immunotherapy. Peripheral blood was partly also available at time points during immunotherapy.

|  |  | **Control** | | **Audencel treatment** | |
| --- | --- | --- | --- | --- | --- |
| **NUMBER OF  SAMPLES ANALYZED** | | **Before** | **During** | **Before** | **During** |
| **Peripheral blood** (PBMC/DC) | ELISPOT | 4 | NA | 32 | 22 |
|  | FACS | 7 | NA | 43 | 34 |
|  | CBA | 4 | NA | 36 | 26 |
|  | RT-PCR | 7 | NA | 43 | 34 |
| **Tumor** | IHC | 14 | NA | 11 | NA |
|  | TCR | 15 | NA | 23 | NA |

Note:
Before = peripheral blood taken from apheresis (treatment group) or from venipuncture (control group), tumor taken from initial surgery
During = peripheral blood taken from venipuncture in weeks 12,26,39,52,65,78,91,104, as far as the patient was alive

**Table S2.** Overview of all variables measured in samples *before* immunotherapy (Audencel-treated patients only) and the respective results of statistical testing.

|  | **Overall survival (OS)** | **Progression-free survival (PFS)** |
| --- | --- | --- |
| **Significant association with survival in Pearson correlation *and* Kaplan-Meier analysis** | *ELISPOT:* Granzyme B *FACS:* CD8+, Monocytes | *ELISPOT:* IFNγ |
| **Significant association with survival in Pearson correlation** | *ELISPOT:* IFNγ *FACS:* Tregs (↓), Term. Dif. CD8+, Plasma blasts *RT-PCR:* %Th1 | *FACS:* Monocytes, CD314 (NKG2D), CD8+B7H1+, CD4+B7H1+ *IHC:* CD3 overall/perivascular, CD8 overall/central tumor, CD4 margin/perivascular, CD45RO overall/margin, CD31 margin |
| **No significant association with survival** | *FACS:* CD3+ cells, CD4+ cells/subsets, CD8+ subsets, B cells/subsets, NK cells/subsets, NKT cells, Granulocytes, MDSCs, Th17  *CBA:* IL-17, IFNg, TFNa, IL-10, IL-6, IL-4, IL-2  *RT-PCR:* IFNγ, Tbet, IL-4, GATA3, IL-17, ROR-γt, IL-10, FOXp3, TGFβ, Th1, Th2, Th17, Treg, %Th2, %Th17, %Treg *IHC:* CD3, CD8, CD31, CD4, CD45RO, p53 | *FACS:* CD3+ cells, CD8+ cells/ subsets, CD4+ cells/subsets, B cells/subsets, NK cells, Tregs/subsets, Granulocytes, MDSCs, Th17 *CBA:* IL-17, IFNg, TFNa, IL-10, IL-6, IL-4, IL-2  *RT-PCR:* IFNγ, Tbet, IL-4, GATA3, IL-17, ROR-γt, IL-10, FOXp3, TGFβ, Th1, Th2, Th17, Treg, %Th1, %Th2, %Th17, %Treg *IHC:* p53 |

Note: all correlations are positive unless otherwise indicated via the sign ↓.

**Table S3.** Overview of all variables measured in peripheral blood samples *with every cycle* of immunotherapy and the respective results of statistical testing.

|  | **Peripheral blood immunovariables measured repeatedly with every cycle of immunotherapy** |
| --- | --- |
| **Significant correlation with the number of vaccines administered (Pearson correlation)** | *ELISPOT:* IFNγ *FACS:* CD4+ cells (↓), CM CD4+ cells (↓), Naive CD4+ cells (↓), EM CD4+ cells (↓), Early Dif. CD4+ cells (↓), Tregs (↓), Th17 (↓)  *CBA:* IL-17, IL-2  *RT-PCR:* IFNg (↓), Tbet, Th1 |
| **No significant correlation with the number of vaccines administered** | *ELISPOT:* Granzyme B  *FACS:* CD3+ cells, CD8+ cells/subsets, B cells/subsets, NK cells/subsets, Th17 cells, Granulocytes, Monocytes, MDSCs  *CBA:* IFNγ, TNFα, IL-10, IL-6, IL-4  *RT-PCR:* IL-4, GATA3, IL-17, ROR-γt, IL-10, FOXp3, TGFβ, Th2, Th17, Treg, %Th1, %Th2, %Th17, %Treg |

Note: all correlations are positive unless otherwise indicated via the sign ↓.

**Table S4.** Overview of all variables measured *after* immunotherapy (variable value after = average of all available time points after at least one vaccination) and the respective results of statistical testing when combining them with survival measures.

|  | **Peripheral blood immunovariables measured repeatedly with every cycle of immunotherapy & relation to survival** | |
| --- | --- | --- |
|  | **Overall survival (OS)** | **Progression-free survival (PFS)** |
| **Significant association with survival in Pearson correlation *and* Kaplan-Meier analysis** | *ELISPOT:* INFγ  *FACS:* CD8+ cells, CM CD8+, Late dif. CD8+, pre-switched memory B cells, Naive B cells, activated NKT, activated NK, Th17 (↓), Monocytes  *CBA:* IL-10 (↓) | *ELISPOT:* Granzyme B  *FACS:* Naive Tregs (↓), Memory Th17  *CBA:* IL-2 |
| **Significant association with survival in Pearson correlation** | *ELISPOT:* Granzyme B  *FACS:* Transitional B cells, CD8+B7H1+, CD4+B7H1+  *CBA:* IL-17, TNFα, IL-6  *RT-PCR:* IL-17, %Th17 | *ELISPOT:* IFNγ  *FACS:* CD56+CD16-, CD4+B7H1+, Monocytes  *CBA:* TNFα, IL-10, IL-6  *RT-PCR:* IL-17, %Th2 |
| **No significant association with survival** | *FACS:* Tregs/subsets, Th17 subsets, Granulocytes, MDSCs  *CBA:* IFNγ, IL-4, IL-2  *RT-PCR:* IFNγ, Tbet, IL-4, GATA3, RORγt, IL-10, FOXp3, TGFβ, Th1, Th2, Th17, Treg, %Th1, %Th2, %Th17, %Treg | *FACS:* CD3+ cells, CD8+ cells/subsets, CD4+ cells/subsets, B cells/subsets, NK cells/subsets, Tregs, Granulocytes, MDSCs  *CBA:* IFNγ, IL-4  *RT-PCR:* IFNγ, Tbet, IL-4, GATA3, RORγt, IL-10, FOXp3, TGFβ, Th1, Th2, Th17, Treg, %Th1, %Th17, %Tregs |

Note: all correlations are positive unless otherwise indicated via the sign ↓.

**Supplementary Materials and Methods**

*General methodology*

To reach the maximum possible number of samples for the exploratory immunobiological studies described here, all patients receiving Audencel under the trial protocol (and with material available) were studied – even if they surpassed the age limitation given for regulatory efficacy assessment.

*Immunotherapy: Preparation of Audencel DC Vaccine and Quality control Evaluation*

Tumor specimens were removed during surgical resection and stored immediately at 4oC and transported to our GMP (Good Manufacturing Practice) facilities under sterile conditions to generate autologous tumor lysate. Tumor tissue was disrupted mechanically and the fragments lysed by five freeze/thaw cycles. Particulate components were removed by centrifugation. Protein concentration of each tumor lysate was determined by Bradford assay and the vials containing protein lysate were kept frozen at -80oC. Peripheral blood mononuclear cells (PBMCs) were obtained by leukocyte apheresis followed by elutration for the selective enrichment of clinical-scale monocytes. Monocytes were cultured in vitro in Cellgro medium (CellGenix Technology, Freiburg, Germany) with the presence of (317U/ml) recombinant human interleukin-4 (IL-4; CellGenix Technology, Freiburg, Germany) and (1000U/ml) recombinant human granulocyte macrophage-colony stimulating factor (rhGM-CSF; CellGenix Technology, Freiburg, Germany). On day 3, fresh medium containing the same cytokines, at the same concentration was added. On day 6, immature dendritic cells were incubated with autologous tumor lysate together with the immunological adjuvant Keyhole Limpet Hemocyanin (KLH; Calbiochem, Darmstadt, Germany) for 2 hours prior maturation stimulus. Subsequently the DCs were incubated with LPS (200U/ml) and IFNy (50ng/ml) for 6 hours to induce functional maturation. DCs were then harvested and washed with Phosphate Buffer Saline (PBS) (Hyclone, ThermoScientific,Utah, USA). Two aliquot of each vaccine batch were used for quality control that included tests for viruses, mycoplasma, and bacteria by the appropriate clinical procedures. In addition, functional potency and the phenotype of the tumour lysate-loaded DCs was examined in vitro. The purity and phenotype of each DC lot was determined by flow cytometry. Cells were stained with antibodies against CD45, CD3, CD19, CD56, CD14, Major Histocompatibility Complex (MHC-I and –II), CD1a, CD83, CD80 and CD86. Release criteria were more than 70% viable dendritic cells and more than 60% CD86+/ human leukocyte antigen (HLA) DR+/CD80+/CD83+ expression. The DC vaccine for each patient was then kept frozen until application. At the time of treatment an aliquot of the DC cancer vaccine containing approximately 5 million DCs was thawed and inoculated to the corresponding patient by injection intranodally into a tumor-free lymph node.

*Immunotherapy: Treatment schedule*

All patients received the first line standard therapy for GBM: surgery, radiotherapy, and chemotherapy (Temozolomide). Randomization was done after surgery; patients in the treatment arm who received Audencel as an add-on to the standard treatment underwent leukocyte apheresis within 7-14 days after surgery. The first 4 immunizations were administered in weeks 7-10. Six more immunizations were applied in between the 6 blocks of maintenance chemotherapy. After completion of that schedule, patients received boost immunizations every 3 months. The vaccine was applied intranodally; each vaccine aliquot of Audencel contained 1-5 x 10^6^ DC. The immunization schedule continued unaltered even if patients suffered disease recurrence and Temozolomide was withdrawn and replaced with an alternative therapy such as Bevacizumab. Patients of both groups received supportive care for acute or chronic toxicity whenever indicated.

*Blood: Preparation of blood samples for characterization*

Patient blood samples were collected in sodium heparin tubes and peripheral blood mononuclear cells (PBMCs) were separated by Ficoll density gradient centrifugation. PBMCs were washed and stored frozen in freezing media containing 10% dimethyl sulfoxide (DMSO) in our Nitrogen (N2)-Banks upon further in vitro analysis.

*Blood: Cell surface marker analysis of PBMCs*

PBMCs collected at the various time points were thawed at once for each patient in pre-warmed 1x C.T.L. wash media (C.T.L., Europe, Bonn, Germany), washed with PBS, blocked with MOP-21 for 10 min prior to staining for 30 min, at 4° C in the dark with the following cocktails (Supplementary Table 1): T cells (CD3/8/4/27/28/45RA/CCR7), B cells (ID, 24/19/38/27/20/3), NK/NKT cells (CD94/314/HLADR/16/56/8/3), Tregs/Th17 (161/25/4/45RA/127/8/3), Activated/Modulatory cells (4/38/HLADR/8/3/274) and MDSCs (CD11b/HLADR/33/15/14). Cells were washed with fluorescence activated cell sorting (FACS) buffer and stained with 4',6-diamidino-2-phenylindole (DAPI) (Hoechst 33342, Thermo Fischer Scientific, Inc, Waltham, MA, USA) for assuring acquisition of only viable cells. Stained cells were acquired in a BD LSR-II cytometer (BD Bioscience, Heidelberg, Germany) and analyzed by Data-Interpolating Variational Analysis (DIVA) software (BD Bioscience, Heidelberg, Germany).

*Blood: ELISPOT assays of PBMCs*

MAHAS4510 Millipore 96 well plates were pre-wet with 35% ethanol. Wells then were washed three times with PBS, afterwards coating antibodies were added: 1/D1K(IFNg) (MabTechAB, Nacka Strand Sweden) and GB10 (GranzB) ( MabTechAB, Nacka Strand Sweden) at a concentration of 1µg/ml and incubated overnight at 4° C. Plates were then blocked with 5% Human AB Serum in PBS and washed with PBS previous cell plating. PBMCs at a concentration of 50,000/ well were plated and tested for the IFNγ and GranzB production after exposure with or without tumor proteins loaded in autologoues dendritic cells. PBMCs stimulated with PMA/ionomycin were used as technical positive control. After incubation for 20 hours, the plates were washed thoroughly with 0,05% tween-PBS to remove cells from the membrane in the bottom of the plate and biotinylated Abs IFNg (7-B6-1-biotin) (MabTechAB, Nacka Strand, Sweden) and GranzB (GB11 biotin) (MabTechAB, Nacka Strand, Sweden) were added and incubated at a concentration of 1µg/ml for 2 hours at 37oC. Plates were washed and developed with streptavidin-alkaline phosphatase (1mg/ml) (MabTechAB, Nacka Strand Sweden) for 1 hour at room temperature in the dark. After washing, substrate (5-bromo-4-chloro-3-indolyl-phosphate/nitro blue tetrazolium, Sigma-Aldrich Co. LLC, St. Louis, Missouri, USA) was added, incubated until the spots were visible (~30 min) and then washed with tap water. The ELISPOT plates were dried out and read in the ImmunoSpot® S6 Core ELISPOT analyzer (C.T.L., Shaker Heights, OH, US).

*Blood: Real Time RT-PCR of PBMCs*

Total RNA was isolated from PBMCs using AllPrep DNA/RNA/Protein Kit (Qiagen, Germantown, MD, USA) according to manufacturer’s guidelines and concentration and purity were measured. Complementary DNA (cDNA) was generated using MultiScribe Reverse Transcriptase (Life Technologies by Thermo Fischer Scientific, Inc, Waltham, MA, USA), random primers and deoxiribonucleotide (dNTPs) (Life Technologies by Thermo Fischer Scientific, Inc, Waltham, MA, USA) in retrotranscriptase (RT) buffer containing RNAse inhibitor (Life Technologies by Thermo Fischer Scientific, Inc, Waltham, MA, USA). Total cDNA was diluted 1:10 and used as template in the real-time quantitative TaqMan® PCR. Real time PCR runs were done in 96-well optical reaction plates(Life Technologies by Thermo Fischer Scientific, Inc, Waltham, MA, USA) in duplicates, each containing TaqGold® Polymerase (Life Technologies by Thermo Fischer Scientific, Inc, Waltham, MA USA), 1xPCR TaqMan buffer A (Applied Biosystems), dNTPs (Life Technologies by Thermo Fischer Scientific, Inc, Waltham, MA, USA) and each of the corresponding gene expression assays (Applied Biosystems): IFNγ, TBET, IL4, GATA3, IL10, FOXP3, TGFβ, IL17A, RORγT and the housekeeping gene 18ribosomal RNA (18rRNA) in a final volume of 20µl, using a Taqman® 7500 Violet PCR system (Applied Biosystems). Amplification cycles were as follows: 2 min at 50° C, then 10 min at 95° C followed by 45 cycles of 15 seconds at 95° C and 1 min at 60° C. The relative expression of each gene was calculated with the following formula: 2^( -delta delta Ct ) relative to the expression of the housekeeping gene 18rRNA.
